# Age-Dependent Variation in Longevity, Fecundity and Fertility of Gamma-Irradiated *Bagrada hilaris* (Hemiptera: Pentatomidae): Insights for a Sustainable SIT Program

**DOI:** 10.3390/insects16040408

**Published:** 2025-04-13

**Authors:** Alessandra Paolini, Sergio Musmeci, Chiara E. Mainardi, Chiara Peccerillo, Alessia Cemmi, Ilaria Di Sarcina, Francesca Marini, René F. H. Sforza, Massimo Cristofaro

**Affiliations:** 1 Biotechnology and Biological Control Agency (BBCA), Via Angelo Signorelli 105, 00123 Rome, Italym.cristofaro55@gmail.com (M.C.); 2 ENEA, Casaccia Research Center, SSPT-AGROS-Agriculture 4.0 Laboratory, Via Anguillarese 301, 00123 Rome, Italy; 3 Department of Environmental Biology, University of Rome “La Sapienza”, 00185 Rome, Italy; 4 Center of Agriculture, Food and Environment (C3A), University of Trento, 38010 San Michele all’Adige, Italy; 5 ENEA, Casaccia Research Center, NUC-IRAD-GAM Laboratory, Via Anguillarese 301, 00123 Rome, Italy; 6 USDA-ARS-EBCL (European Biological Control Laboratory), 810 Avenue du Campus Agropolis, 34980 Montferrier-sur-Lez, France

**Keywords:** sterile insect technique, gamma irradiation, sterility, biological control, insect pest

## Abstract

*Bagrada hilaris* is an invasive stink bug causing important yield losses in Brassica crops. It originates from India, Southeast Asia, the Middle East and South Africa and is reported as invasive in several southwestern US states, Hawaii, Mexico, Chile and in the Mediterranean islands of Malta and Pantelleria (Italy). In this study, we tested the effects of gamma rays on the longevity, fecundity and fertility of bagrada bugs. We irradiated them at two different stages of their life cycle (fifth-instar nymphs and two-week-old adults). Irradiation at the nymphal stage had a strong impact on female fecundity, with egg numbers approaching zero at a dose of 80 Gy. Similarly, a full suppression of female fertility was achieved at 80 Gy when they were mated with males irradiated as nymphs or as mature adults. For longevity, gamma rays had only a slight impact on adult male and female life span. Due to the evidence of a gregarious phase during the autumn, these results suggest that small-scale SIT-localized applications by massive collections of bagrada bugs at various stages of development during autumn, followed by irradiation and reintroduction to the field, might be a safe and economically sound approach of control. Research is currently underway to evaluate the fitness of sterile males and mating patterns. Further studies in confined-field conditions will be needed.

## Simple Summary

Controlling alien insect pests in cropping systems without chemicals is challenging. Here, we evaluate the use of irradiation to determine the feasibility of the sterile insect technique (SIT) approach to controlling the bagrada bug, *Bagrada hilaris*. This work complements previous investigations carried out on two of the major pentatomid pests, e.g., the brown marmorated stinkbug and the bagrada bug. Due to the evidence of a gregarious phase during the autumn, we tested the possibility of making massive collections of wild-type bagrada bugs, irradiating and releasing them for small-scale SIT programs. This study documented how irradiation doses of gamma rays impact the physiological parameters, such as fertility, fecundity and longevity, of two-week-old bagrada adults and fifth-instar nymphs. Our results warrant further research to test the feasibility of SIT directly on bagrada bug populations in confined-field conditions alone or in combination with a classical biological control program.

## 1. Introduction

Bagrada bug, *Bagrada hilaris* (Burmeister) (Hemiptera: Pentatomidae), is an invasive stink bug species that has been reported as a pest of Brassica crops in Africa, India and the Middle East since the early 1900s [1,2,3]. In the Old World, it is currently widely distributed throughout its native range but also in Southeast Asia and in southern Europe, where it is recorded in the Mediterranean islands of Malta and Pantelleria (Italy) [2,4]. In 2008, it was first reported in southern California and it is now rapidly expanding its range throughout several southwestern US states, Hawaii and Mexico. Recently, it has been recorded in Quilicura, Chile [2,5,6].

*Bagrada hilaris* is a polyphagous pest, infesting a large number of plant species, including crops, weeds and ornamentals belonging to 23 families [2]. Though most of the economic damage occurs in the family Brassicaceae, it was reported also on wheat, corn, legumes [7,8,9] and on capers in the island of Pantelleria (Italy) [10,11]. On brassica crops and capers, the bagrada bug feeds mainly on tender tissues, such as cotyledons and young leaves or flower buds, causing chlorotic lesions that may become necrotic and reduce plant growth or lead to rapid plant death [2,11,12,13].

Currently, few effective alternatives to protect crops from *B. hilaris* are available. Cultural control practices, like the removal of any host plants in crops and adjacent fields, together with field sanitation (removal of fallen vegetables after harvesting), can be useful to prevent *B. hilaris* spreading [2,11] but are expensive, laborious and time consuming. Chemicals, such as pyrethroids and neo-nicotinoids, are commonly used to prevent *B. hilaris* outbreaks in invaded areas but broad-spectrum insecticides are a short-term solution, and their frequent use can induce insecticide resistance. For these reasons more sustainable methods for the economy and the environment are needed [12,14].

Classical biological control, based on the introduction of exotic biocontrol agents or enhancement of indigenous ones have shown promising medium- and long-term outcomes controlling bagrada bugs [15]. Some egg parasitoid wasps have been identified and tested as potential biocontrol agents of bagrada bugs [2,16,17]. Among them, the egg parasitoid *Gryon aetherium* Talamas (formerly *G. gonikopalense* (Sharma)) (Hymenoptera: Scelionidae) has been selected as the most promising candidate because of its relative host-specificity and its capability to detect and oviposit on buried bagrada bug eggs [18,19].

Integrated pest management (IPM) can include classical biological control (CBC) in combination with sterile insect technique (SIT), as components for a synergistic and effective program. SIT is a species-specific method, and it relies on the mass production, sterilization and subsequent inundative release of predominantly sterile male insects [20]. The additive effect of the combination between the SIT and the classical biological control [21,22] further strengthens the opportunity to implement IPM or area-wide eradication programs for managing *B. hilaris* within its invasive range [22,23,24].

A pest management program that involves the use of biological control agents (BCAs) requires an effective field monitoring system for their detection, which, for stink bug pests, is based on sentinel eggs for egg parasitoids [25]. To minimize the risk of inadvertently introducing the pest into the environment by using fertile sentinel eggs, several alternatives were evaluated, such as unfertilized and refrigerated eggs. Recently, Cristofaro et al. [26] and Roselli et al. [27] proposed a new concept of using sterile sentinel eggs produced by the SIT for the management of pentatomid pests since wild fertile females mating with irradiated males laid sterile eggs, which are a suitable (and long-life) substrate for the oviposition and the full larval development of egg parasitoids.

Both pentatomid pest species, *B. hilaris* and *Halyomorpha halys* (Stål) display a gregarious behavior before and during the winter diapause, which could be an opportunity to obtain “wild-type” competitive stink bug adults to irradiate and release in small pilot SIT program contexts [24,26,28].

Previous studied showed the promising effects of the irradiation using gamma rays on the longevity, fecundity and fertility of newly emerged adults of *B. hilaris* [26]; to further the possibility of using field collected stages instead than build bio-factories, here we present a study on the effects of gamma rays on the same physiological parameters when irradiation has been conducted on mature adults and on nymph instars.

## 2. Materials and Methods

### 2.1. Insect Collection and Colony Maintenance

During the late summer and the autumn 2023, three field surveys were carried out for collecting aggregating adults and nymphs of *B. hilaris* on caper plants (*Capparis spinosa* L.) in the locality of Scauri (36.7722; 11.0606; 22 m a.s.l.) on Pantelleria Island, Italy. Living material was transferred to the quarantine facilities of Edmund Mach Foundation, San Michele all’Adige, Trento, Italy.

Insects were reared in BugDORM^®^ cloth cages (30 × 30 × 30 cm, 680 μm opening mesh) at 26–22 °C (day-night) with a 14:10 h (L:D) photoperiod and 50–60% RH [29].

Brussels sprouts (*Brassica oleracea* L. var. *gemmifera*) were provided as food source and refreshed three times per week. When unavailable, they were replaced with Savoy cabbage (*B. oleracea* L. var. *sabauda*) or, less frequently, green cabbage (*B. oleracea* L. var. *capitata*) cuttings.

A plastic Petri dish of 9 cm diameter, filled with sand, was placed in each cage as oviposition substrate.

Field collected insects were supplemented periodically to the colony to maintain genetic diversity.

### 2.2. Selection of Physiological Stages

Since the purpose of the work was to evaluate the effects of gamma rays on the longevity, fecundity and fertility of field collected bagrada bugs, it was necessary to standardize the age of the insects utilized for the bioassays. For this reason, following previous histological observations (Dallai and Paoli, Pers. Comm.) on *H. halys* and on *Nezara viridula* (L.) [30], we decided to irradiate insects at the fifth nymphal stage because at that physiological stage the gonad development process has already started. Nymphal stage was detected according to the morphological characters indicated by Azim and Shafee (1986) [31]. For mature adults, it was not possible to screen for the effects of irradiation on “wild-type” adults because we could not assess the age of these adults and so could not estimate the effects of the irradiation on longevity. For this reason, starting from “wild-type” nymphs collected in the field, we reared them until the adult stage and we irradiated adults when they were two weeks old (in captivity conditions the longevity of *B. hilaris* ranges between 21 and 28 days [26]).

### 2.3. Irradiation and Experimental Design

Insects were irradiated at the Calliope gamma facility (^60^Co source, [32]) at ENEA (Italian National Agency for New Technologies, Energy and Sustainable Economic Development) Casaccia R.C. in Rome. The irradiation tests were performed at a mean dose rate around 160 Gy/h (2.67 Gy/min). The dose rates were determined by experimental Fricke dosimetry and the correspondent values were referred to water. The measurements took into consideration the sample size, and therefore five dosimeters were used for each test. This procedure ensures a dose uniformity of 5%.

#### 2.3.1. First Experiment

In the first experiment, fifth-instar nymphs were exposed to doses of 32, 40, 60, 80 and 100 Gy. The starting irradiation dose of 32 Gy was selected according to previous studies carried out on *H. halys* and *B. hilaris* [26,28]. Irradiated nymphs were then placed singularly in Petri dishes (9 cm Ø) with food to obtain virgin adults for the experiment. The same procedure was applied to the insects in the control (0 Gy), and they were both (treated and control) exposed to the same holding conditions as the colony.

As soon as they reached the adult stage, virgin adults were tested through the following crosses:

1. Irradiated male x Healthy female (NyIM/HF).
2. Healthy male x Irradiated female (HM/NyIF).
3. Healthy male x Healthy female (HM/HF) (control).

Crosses were set up in transparent 500 mL glass jars (12 cm diameter, 5 cm h) confining each pair with one Brussels sprout as the food source. One small Petri dish (5 cm Ø) filled with sand was placed in each jar for oviposition.

The pairs were checked every 2–3 days to record mortality and the Petri dishes were inspected under a stereomicroscope (Olympus SZX-ILLB200, Hamburg, Germany) to count oviposited eggs. Petri dishes with eggs were maintained at the same environmental conditions to evaluate the eclosion rate. Tested adults that survived after the experiments were kept until death to evaluate longevity.

#### 2.3.2. Second Experiment

In the second experiment, gamma irradiation at doses of 60, 80, 100 and 120 Gy was applied to two-week-old males taken from the rearing cages. Virgin females were instead obtained confining fifth-instar nymphs singularly in Petri dishes (9 cm Ø) with food.

Adults obtained were crossed as follows:

1. Old irradiated male x Healthy female (OIM/HF).
2. Healthy male x Healthy female (HM/HF) (control).

As in experiment 1, each pair of insects was confined in a 500 mL (12 cm diameter, 5 cm h) transparent glass jar, providing one Brussels sprout as the food source and a 5 cm diameter plastic Petri dish filled with sand for the oviposition site. Mortality and eggs oviposited were registered every 2–3 days as well as the hatching of the eggs.

### 2.4. Statistical Analysis

#### 2.4.1. Data Modeling on Reproductive Parameters

Generalized Linear Models (GLiMs) [33] were performed to assess the effect of gamma irradiation on the response variables number of hatched eggs, number of laid eggs and rate of hatched eggs. The GLiM model was chosen after the assessment of a significant deviation from normality by the Shapiro–Wilks test and after an analysis of fit, residuals and a q–q plot. The predictor variables “type of bug cross” (discrete) and “dose Gy” (continuous) were used in the model with a quasi-Poisson distribution and with a log*_e_θµ_i_* link function, where *µ_i_* was the mean expected response and *θ* was the estimated overdispersion parameter. In the case of the percentage of hatched eggs, a logarithmic transformation was applied to stabilize variance [33]. The models were fitted to assess whether (1) there was a relationship between the irradiation dose and the response variables and (2) if the different levels of the categorical predictor had different slopes, thus indicating a differential effect of the gamma rays on the different crosses.

Two analyses were performed on two separate groups: (1) males and females emerged from irradiated nymphs and (2) males irradiated at 3 different ages of development: (a) at the nymphal stage (NyIM); (b) immediately after adult emergence (NEIM); and (c) after about two weeks from adult emergence (OIM). In the case of the second analysis, we compared the data of group “b” (NEIM), recently published [26] with the new results obtained testing insects belonging to groups “a” (NyIM) and “c” (OIM).

In both the analyses the dose effect was treated as a covariance term, since the untreated group was considered as the zero dose of the treatments, thus allowing the different groups to keep their own intercept so as to avoid aliased terms. In the case of group 1 (the females and males emerged from irradiated nymphs), a two-sample Wilcoxon test was performed in order to compare the effects of gamma irradiation at specific doses (stats package, R statistical Functions, Version 3.6.2), while in the case of group 2, a Dunn test for mean separation (FSA package, Simple Fisheries Stock Assessment Methods, Version 0.9.4) was performed to compare the 3 types of males irradiated at different ages of development. When means values are reported in the text, they are always followed by the standard errors.

#### 2.4.2. Data Modeling for the Dose–Response Estimation in Egg Hatching

A curve model was applied with functions drm and mselect in the drc package (Dose–Response Curves, Version 3.0-1) [34] to define the effective dose values in the different types of crosses. The best model was chosen by comparing the log-likelihood values, Akaike’s Information Criteria (AIC), lack of fit and residual variance of all models. The % of response of egg hatching was compared to the control and was considered as the response variable. This was expressed as Pt/Pc × 100 (Abbott correction [35]), where Pt is the proportion of hatched eggs for the treatment and Pc is the proportion of egg eclosion for the control. The dose treatment (treated as a continuous variable) and the type of crosses (treated as categorical variables) were the explanatory variables. In the case of males, the dataset was fitted to a non-linear Weibull model, chosen after the analysis of diagnostic parameters described above:

W1.3 curve    y = *c* + (*d* − *c*) − exp {−exp{*b*[log(x) − log(*e*)]}}. (1)

For this model, *d* is the upper limit, *c* is the lower limit, *b* is the slope, *e* is the inflection point and x is the irradiation dose (Gy). The *c* parameter (the lower limit) was shared at zero among the 3 treatments (W1.3 model), as it was assumed there were no eclosed eggs for the values of dose irradiation for lim f(x) → ∞. The *d* parameter was also shared among the three types of crosses, since no differences were assumed for the three types of crosses at dose 0. The distribution of errors was corrected by the Box–Cox transformation. In the case of the number of hatched eggs, the same Weibull model was also applied for plotting the curve trends.

For group 1 (the comparison between males and females irradiated at the nymphal stage), a LL2 curve model was performed on females. This choice was made based on AIC and the other diagnostic parameters described above. Although the exponential decay curve showed similar AIC values, the LL2 curve was considered more likely, assuming some degree of resilience to low irradiation exposures which is common in most biological systems. Particularly, two logistic curves were selected: the LL2.3 curve in the case of the % of response in egg hatching and the LL2.4 curve for the number of hatched eggs.

LL2.3 curve    y= *c* + (1 − *c*)/{1 + exp{*b*[log(x) − *e*]}} (2)

LL2.4 curve    y= *c* + (*d* − *c*)/{1 + exp{*b*[log(x) − log(*e*)]}} (3)

The parameters *b*, *c*, *d* and *e* describe the same functions as described in the Weibull model.

Due to the high degree of uncertainty found in the curve parameter estimates performed on the irradiated females, sterilizing effective doses (EDs) were calculated only in the case of group 2 (the irradiated males at 3 different ages of development). Sterilizing effective doses ED50, ED90, ED95 and ED99% were determined with their respective 95% confidence intervals. Statistical significance of the treatment (the type of cross) was verified by comparing the whole model without treatments with that of treatments using an ANOVA simple test by the anova.drc function, while the comparisons among groups for model parameters and ED values were performed by the compParm and EDcomp functions, respectively. All graphs were constructed using the plot function in the graphics package [36] in the statistical environment R [37].

#### 2.4.3. Survival Analysis on Adults

A survival analysis was performed using the R package survival (a package for survival analysis version 3.7-0) [38,39] on data regarding adult longevity. In this case, only the NyIM/HF and the OIM/HF groups were considered in the analysis, as in the NEIM/HF group was not suitable for an adequate estimate and data sampling was made with censored data and was considerably smaller in comparison to the other two crosses. The presence of groups of individuals with different life spans was analyzed by frequency histograms and a Gaussian Kernel smoother was used [40]. Kernel density plots of frequency were performed by the geom_density function in ggplot2 package (version 3.3.3) [41]. The Kaplan–Meyer survival curves were analyzed by the log-rank test (Mantel–Cox). In addition, a GiLM was applied on longevity data according to a full factorial design, to investigate if a differential effect of irradiation was present between the treatments. Since longevity shows high overdispersion, a quasi-Poisson model with a log*_e_θµ_i_* link function was used. In experiment 1, performed on individuals irradiated at the nymphal stage, the longevity of irradiated adult males was compared with that of irradiated females. Thus, sex was considered as an explanatory variable while the second explanatory variable was the applied dose of irradiation, the latter treated as a categorical factor. The second experiment, performed on the irradiated males at nymphal stage and at the late adult age, was analyzed according to the same experimental design. In the case of the first experiment, the 0-, 32-, 60-, 80- and 100-dose Gy were analyzed, while in the second one, only the doses 0, 60, 80 and 100 were compared. Finally, a Dunn test for mean separation was applied within each treatment to easily compare the effects recorded at specific doses of irradiation. All the experiments were terminated with the death of the adults.

## 3. Results

### 3.1. Effects of Gamma Irradiation on Oviposition and Egg Eclosion After Crosses with Males or Females Emerged from Irradiated Nymphs

#### 3.1.1. Fecundity

Gamma irradiation had a strong effect on female fecundity, heavily impairing oviposition already starting from the lowest dose of 32 Gy. The number of laid eggs per female was only 10.5 ± 3.1 at this dose compared with the 150.7 ± 13.6 eggs recorded under the untreated control (Table 1). The egg number approached zero at 80 Gy (mean of 1.5 ± 0.86). The decline in oviposition with increasing doses was much slower when the cross was performed between untreated females and irradiated males. A weak decline in fecundity started at over 60 Gy. At 100 Gy the number of laid eggs was 75.8 ± 13.2 versus 154.5 ± 12.4 eggs recorded at 0 Gy. These results were confirmed by the GiLM analysis (Table A1). A very strong negative effect of gamma rays was found (*t*-value = −21.56 ***), combined with a strong effect of the interaction term (*t*-value = 16.97 ***), thus suggesting a marked differential effect of irradiation on sexes. The negative effect of gamma irradiation was stronger in the case of irradiated females (NyIF), as the positive *t*-value of the NyIM/HF cross suggests. Particularly, fecundity was even higher at 60 Gy than at 0 Gy in the cross-irradiated males and healthy females (NyIM/HF cross) (175.0 ± 15.1 eggs at 60 Gy and 154.5 ± 12.4 eggs on the untreated), though this difference was not statistically significant.

**Figure 1:**
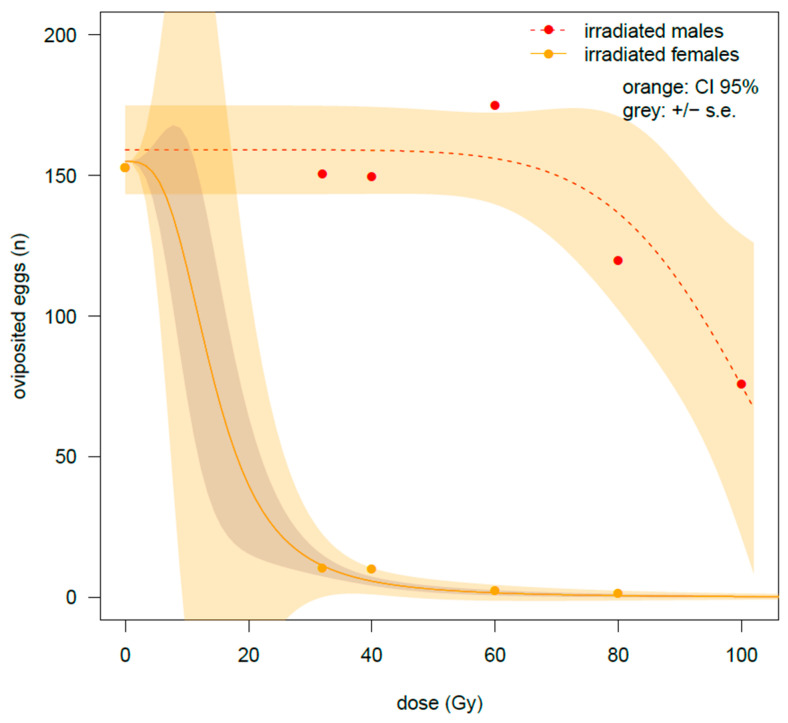
Experiment on male and female adults emerged from late-stage irradiated nymphs. Two types of crosses are compared for the number of oviposited eggs: irradiated males × healthy virgin females or irradiated females × healthy virgin males. Data were fitted by a W1.3 and LL2.3 model for the irradiated males and for the irradiated females, respectively.

The different trends are also reported in Figure 1 where two types of curves have been hypothesized, the LL2.3 for the irradiated females and the W1.3 for the cross with the irradiated males. It is worth to note that in the case of irradiated males, the curve fit was quite poor suggesting caution, particularly in extrapolating the trend based on the curve parameters.

#### 3.1.2. Fertility

For irradiated females at the nymphal stage, the effect of the gamma irradiation on fertility suppression was even higher than on fecundity. A drop in the number of hatched eggs was observed at 32 Gy, when only 1.1 ± 0.9 eggs hatched in comparison to the 97.1 ± 11.8 eggs per female observed at 0 Gy. Fertility was even lower at higher doses, and from 60 Gy upwards, eggs hatched very sporadically, remaining below one hatched egg per female (Table 1).

**Figure 2:**
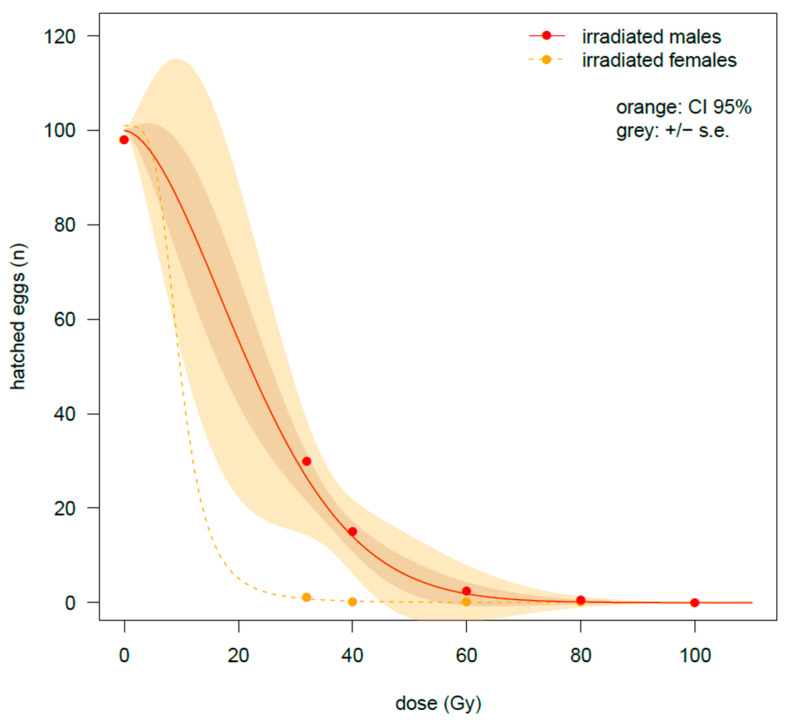
Experiment on males and female adults emerged from late-stage irradiated nymphs. Two types of crosses are compared for the number of hatched eggs: irradiated males × healthy virgin females or irradiated females × healthy virgin males. Data were fitted by a W1.3 and LL2.4 model for the irradiated males and for the irradiated females, respectively. In the case of treated females, due to the high uncertainness at low doses of irradiation, error limits are not shown.

The effect of irradiation on fertility was also strong if the males were irradiated at the nymphal stage. Particularly, fertility of the unirradiated female was reduced by about two-thirds when mated with males irradiated at 32 Gy (Table 1). The number of eclosed eggs dropped to 2.35 ± 1.18 with males irradiated at 60 Gy, then approaching 0 at 80 Gy. Finally, no eclosion was recorded at 100 Gy. As Table A1 shows, a strong effect of dose irradiation was found (*t*-value = −14.64 ***), as well as an interaction effect between dose and NyIM/HF on fertility (*t*-value = 6.30 ***), suggesting a slightly slower effect on female fertility with increasing doses when males were irradiated (positive *t*-value). The difference in trends is reported in Figure 2, where the more pronounced drop in fertility can be noted when the irradiation treatment was applied to the female nymphs in comparison to the irradiated male nymphs. In any case, the suppression of fertility was quite sharp also in the NyIM/HF cross and was almost complete from 60 Gy upwards.

#### 3.1.3. Percentage of Hatched Eggs

**Figure 3:**
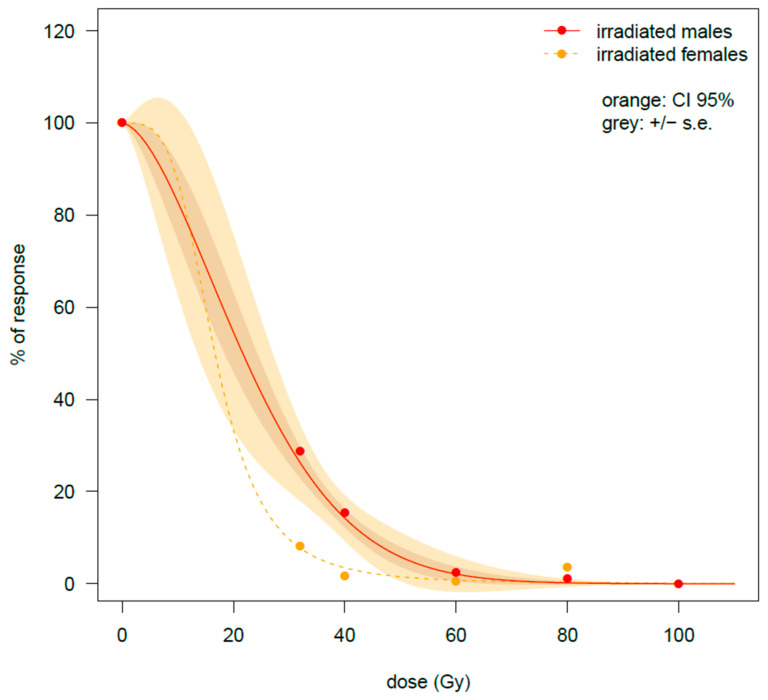
Experiment on male and female adults emerged from late-stage irradiated nymphs. Egg hatching rate after γ irradiation performed on males of *Bagrada hilaris*. The untreated was fixed at 100% by Abbott transformation. Two types of crosses are compared: irradiated males × healthy virgin females or irradiated females × healthy virgin males. Data were fitted by a W1.3 and LL2.3 model for the irradiated males and for the irradiated females, respectively. In the case of treated females, CIs at 95% are not shown, due to the high uncertainness at low doses of irradiation.

As for the fertility tests above, the drop in the hatching rate of irradiated females was drastic, from 58.2% ± 4.04 for the untreated control to 4.8% ± 3.33 observed in the females irradiated at 32 Gy at the nymphal stage. The lowest value was recorded at 60 Gy (0.71% ± 0.71), while an apparent recovery was found at 80 Gy (9.5 ± 9.5), likely by chance, because few eggs were available for the percentage calculation (Table 1). According to the GiLM model (Table A1), a slower drop in the reproductive potential of male nymphs with an irradiation dose was observed compared with females (interaction term *t*-value = 2.982 **). The percentage of egg eclosion was 16.9 ± 2.7% with the males irradiated at 32 Gy in comparison to the 4.8% recorded on the irradiated females. However, when the males were irradiated at 60 Gy at the nymphal stage, eclosion rates were similar to those observed from the cross between health males and irradiated females (HM/NyIF) (1.45% ± 0.59 against 0.71% ± 0.71 recorded on the treated females), approaching zero at 80 Gy (0.62 ± 0.46) and reaching zero at 100 Gy, when no egg eclosion was observed. The trends are visualized in the fitted curves of Figure 3.

Due to the extreme curve steepness between zero and the 32 Gy, curve parameters were highly uncertain and thus not significant for the curve of irradiated females. For this reason, no inferences were made in this case. For the cross between irradiated males at the nymphal stage and healthy females (NyIM/HF), the curve trends of the percentage of response in egg hatching will be analyzed more in depth in the next section.

### 3.2. Effects of Gamma Irradiation on Oviposition and Egg Eclosion After Crosses with Males Irradiated at Three Different Ages of Development

#### 3.2.1. Fecundity

The effects of gamma irradiation were small or negligible, and data showed high variability for this reproductive parameter, with no clear patterns among the three crosses. The cross between newly emerged irradiated males and healthy females (NEIM/HF) showed substantial retention or even enhancement of fecundity at high doses. Particularly, a peak of 206.2 ± 37.8 laid eggs was observed at 80 Gy (Table 2) in comparison to the untreated (129.9 ± 22.0). This value was significantly higher than that recorded on the cross between irradiated old males and healthy females (OIM/HF) at 80 Gy (Dunn test *z* = 2.877, *p*-value = 0.012). In the OIM/HF cross, an apparent decrease in fecundity was observed at 80 Gy, followed by an increase at the tested highest dose of 120 Gy (Table 2). The cross between males irradiated at the nymphal stage and healthy females (NyIM/HF) showed a slight decrease at higher doses (from 154.54 ± 12.45 eggs at 0 Gy to 75.8 ± 13.2 eggs recorded at 100 Gy). The effects were evaluated in the GiLM model (Table A2). The dose effect was weakly significant (Table A2), thus suggesting that, on average, irradiation could induce only a slight suppression of fecundity.

The interaction coefficients (Table A2) were significant only in the case of the crosses between the NEIM/HF and the NyIM/HF groups (*t* = 2.086 *), indicating a possible higher retention of fecundity also applying high doses of irradiation in the cross with newly emerged irradiated males if compared to the males irradiated at the nymphal stage.

#### 3.2.2. Fertility

**Figure 4:**
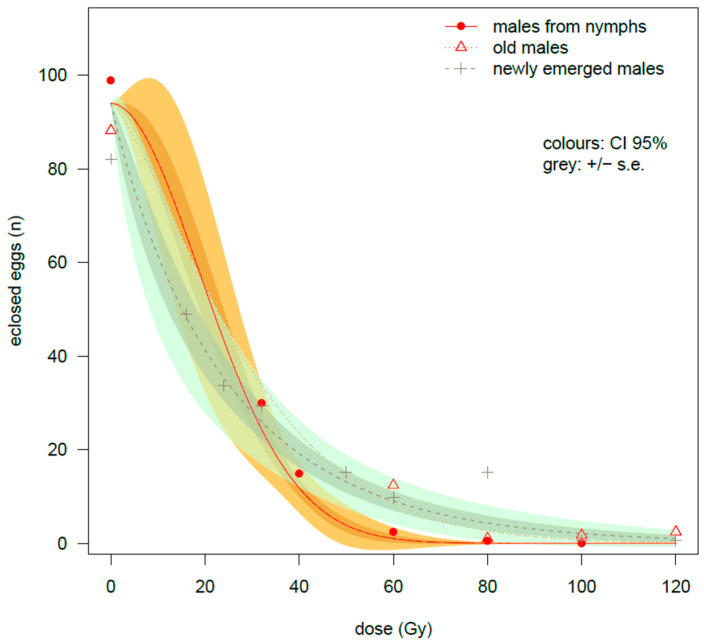
Comparison among males irradiated at 3 different phases of development. Number of eclosed eggs after γ irradiation performed on males of *Bagrada hilaris*. Three different phases of male development are compared: males irradiated at the nymphal stage, newly emerged males and males irradiated after about two weeks from eclosion. Data were fitted by a Weibull curve model (W1.3). The data of newly emerged males were already presented in another paper [26] and are included here for completeness. Error limits are reported only for the males irradiated at the nymphal stage and for the newly emerged irradiated males for clarity.

The GiLM shows (Table A2) that the effect of gamma rays on the suppression of egg fertility was strong in all the three types of cross. However, the cross between males irradiated at the nymphal stage and healthy females (NyIM/HF) recorded a sharper drop in egg hatching (Table A2) in comparison to the other two crosses (interaction term of the NEIM/HF group: *t*-value = 2.573 *; interaction term of the cross OIM/HF: *t*-value = 2.538 *). The number of hatched eggs dropped to 2.45 ± 1.2 at 60 Gy in the NyIM/HF cross (Table 2), while the decrease in fertility was slower in the NEIM/HF cross (9.8 ± 4.8 eclosed eggs) as well as in the OIM/HF cross, the latter recording 12.4 ± 2.5 eclosed eggs (Dunn test comparison NyIM/HF-OIM/HF: *z*-value = −4.13. *p*-value = 0.0001). The difference between the NyIM/HF cross and the NEIM/HF cross was greater at 80 Gy, since the females crossed with the males irradiated at the nymphal stage had virtually no hatched eggs (0.47 ± 0.35 eclosed eggs) in comparison to the 15.2 ± 7.2 hatched eggs obtained from the females mated with newly emerged irradiated males (NyIM/HF-NEIM/HF comparison: *z*-value = −3.66, *p*-value = 0.0007). At the same dose of 80 Gy, the OIM/HF cross had a sharper decrease in eggs hatching (1.10 ± 0.37) than the NEIM/HF cross (Dunn test: *z*-value = 2.67, *p*-value = 0.011), approaching the values of NyIM/HF cross. The overall trend can be observed in Figure 4 where the three Weibull curves are compared.

These curves show different steepness between the two groups of irradiated adults and the group of irradiated nymphs. Particularly, the estimated ratio of effective dose was statistically significant in the comparison between the old irradiated males/healthy females (OIM/HF) and the cross between males irradiated at the nymphal stage and healthy females (NyIM/HF) (at ED90: ratio = 1.382 *), indicating a lesser effect of irradiation at intermediate doses in the cross with the irradiated old males in comparison to the males irradiated at the nymphal stage.

#### 3.2.3. Percentage of Response in Hatched Eggs

Increased exposure to gamma irradiation resulted in a sharper drop in the percentage of eclosion for the NyIM/HF cross in comparison to the other two crosses NEIM/HF and OIM/HF (Table 2). In the case of NyIM/HF treated at 60 Gy, 1.5 ± 0.61% of the eggs hatched, compared with 6.2 ± 3.4% and 8.7 ± 1.6% offspring recorded on the NEIM/HF and OIM/HF crosses (NyIM/HF-OIM/HF Dunn test comparison: *z*-value = −4.338, *p*-value ≤ 0.0001). In the case of the NyIM/HF group, the % of response approached zero at 80 Gy (0.6 ± 0.46%), and no hatched eggs were recorded at 100 Gy. In contrast, the hatching rate remained at 7.5 ± 4.0% in the NEIM/HF cross at 80 Gy (NyIM/HF-NEIM/HF comparison: *z*-value = −3.196, *p*-value = 0.0042). The OIM/HF cross showed a sharper decrease at 80 Gy, in comparison to the NEIM/HF cross, since the percentage of response was 1.26 ± 0.38% at this dose (Dunn test comparison: *z*-value = 2.235, *p*-value = 0.0381). In any case, when the females mated with the irradiated adults, the percentage of response remained at about 1% even if doses of gamma rays greater than 100 Gy were applied (Table 2). These differences are summarized in the table of the GiLM model (Table A2). Beyond the strong effect of the dose, the interaction term coefficients dose × cross were both significant, (OIM/HF coefficient: *t*-value = 4.53 ***; NEIM/HF coefficient: *t*-value = 2.45 *). The positive value of the interaction term indicated a slower decrease in the % of egg hatching with increasing doses if the males were irradiated as adults than as nymphs.

#### 3.2.4. Estimation of the Weibull Curve Model for the % of Response in Egg Hatching

**Figure 5:**
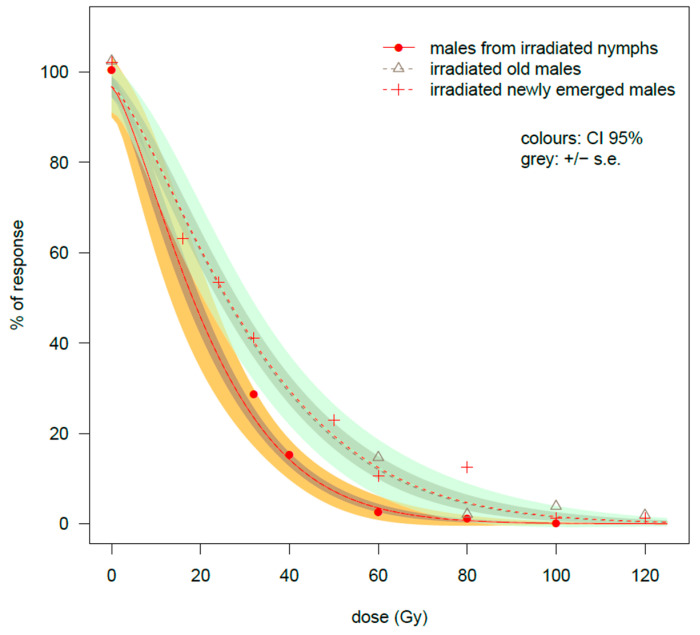
Comparison among males irradiated at 3 different phases of development. Egg hatching rate after γ irradiation performed on males of *Bagrada hilaris*. The untreated was fixed at 100% by Abbott transformation. Three different phases of male development are compared: males irradiated at the nymphal stage, newly emerged males and “old” males irradiated after about two weeks from adult emergence. Data were fitted by a W1.3 Weibull curve model (see Section 3.2.4 for details). The data of the newly emerged males were already presented in another paper [26] and are included here for completeness. Error limits are reported only for the males irradiated at the nymphal stage and for the newly emerged irradiated males for clarity.

The relationship between the % of response in eggs hatching and dose are given in Figure 5 while the estimation of curve parameters is presented in Table 3.

All the parameters had high and strongly significant *t*-values, indicating a good fit of the model to the data. The parameter *b* describes the steepness of curve, while the parameter *d* describes the upper limit: both these parameters were shared among the three types of crosses. Since the parameter *c* was set to zero, only the parameter *e* (the inflection point of the curve) was allowed to vary among the three curves. From Table 3, the difference for the *e* parameter between the NyIM/HF cross (24.77 ± 2.42 Gy) and the two crosses NEIM/HF and OIM/HF (35.58 ± 3.80 and 35.07 ± 4.23 Gy, respectively) was significant for both groups. In fact, the estimated ratios of effective doses were significantly higher than 1 when the crosses with irradiated adults were compared with the NyIM/HF cross (NEIM/HF-NyIM/HF cross: ratio = 1.437 *; OIM/HF-NyIM/HF cross: ratio = 1.416 **), indicating a stronger effect of irradiation on the NyIM/HF cross in both comparisons, while virtually no differences were detected between the OIM/HF and NEIM/HF crosses (ratio = 0.986).

The effective dose estimates are reported in Table 4. A suppression of 95% in the egg hatching response (ED95) was achieved at 54.93 ± 3.98 Gy in the NyIM/HF cross, while an ED95 value of 78.92 ± 9.61 Gy was obtained in the NEIM/HF cross, very close to the 77.80 ± 5.54 dose Gy of the OIM/HF cross. These results indicate that a significantly lower dose was necessary to obtain the 95% of egg sterilization when the males were irradiated as nymphs. At ED99 the estimates were 75.07 ± 7.92 on NyIM/HF, 107.84 ± 16.61 on NEIM/HF and 106.32 ± 10.05 on the OIM/HF cross, confirming the higher sensitivity to radiation exposure of NyIM/HF in comparison to the other two groups. However, due to sporadic cases of egg hatching, the estimates were more uncertain at ED99, especially in the case of NEIM/HF and OIM/HF crosses (see the error limits in Table 4).

### 3.3. Survival Analysis of Adults

#### 3.3.1. Male and Female Adults Emerged from Irradiated Nymphs

In the experiment with insects from irradiated nymphs, the negative effect of irradiation on life span was significant for both sexes (females: score (logrank) test = 16.42, *p*-value = 0.006; males: score (logrank) test = 57.82 on 5 df, *p*-value = 3 × 10^−11^). However, according to the GiLM model (Table A3), a differential effect was found (overall effects of dose: χ^2^ = 561.48, *p*-value = 6.93 × 10^−11^; dose * × sex: χ^2^ = 271.1, *p*-value = 5.21 × 10^−5^).

In particular, the males survived longer than the females at zero or at the lowest dose of 32 Gy, but with increasing doses, the negative effect on longevity was gradually stronger compared to that observed on the females (Table 5), as the interaction terms of the GiLM model show (Table A3 in Appendix A).

In fact (Table 5), no evident trend was found for females with increasing doses and only the dose of 32 Gy was significantly lower than the untreated (25.30 ± 3.19 days against 38.5 ± 1.88 of the untreated). Thus, gamma irradiation negatively affected the longevity of males, and these effects were progressively larger and statistically significant if compared to the untreated starting from 40 Gy upward (29.14 ± 3.61 days at 40 Gy up to 18.71 ± 2.85 days at 100 Gy, compared to the 51.04 ± 3.27 days recorded on the control).

**Figure A1:**
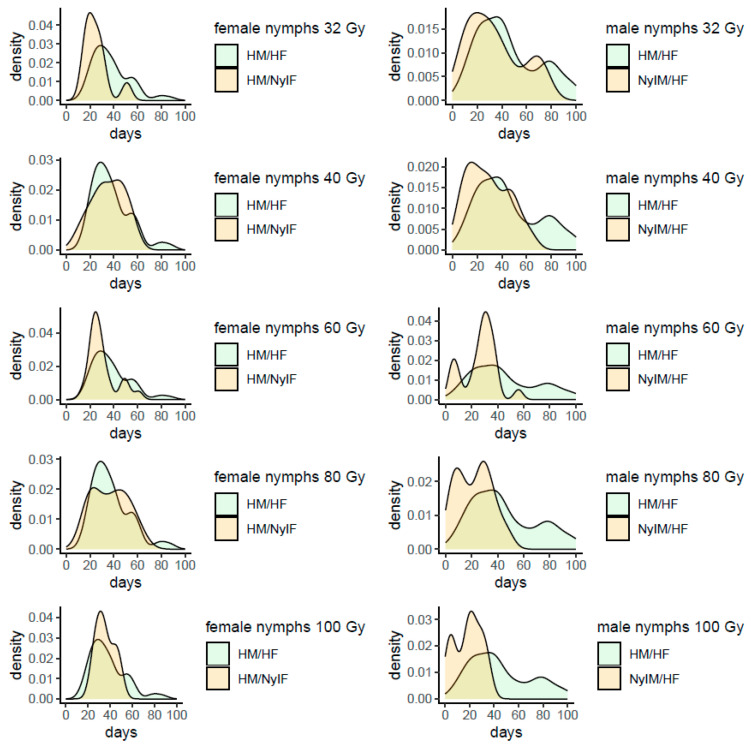
Experiment on male and female adults emerged from late-stage irradiated nymphs: Kernel density plots of frequency. Comparison of the frequency distribution of the life span among the different treatments of irradiation applied on female nymphs (HM/NyIF) or male nymphs (NyIM/HF). The green curves represent the untreated and are compared with the orange curves of the irradiated individuals (HM/HF) at the doses of 32, 40, 60, 80 and 100 Gy.

The Kernel density curves of smoothed frequency of Figure A1 show that on females, irradiation treatments had only a weak effect mainly on the longer-lived individuals, while no further shortening of longevity was observed at doses higher than 32 Gy. In the case of males, the decrease in life span was more evident with increasing doses. However, 50% of males survived at least 3 weeks or more even from 60 Gy upward. Particularly, a peak of frequency at about 30 days was observed at 60 Gy, while at 80 and 100 Gy a portion higher than 50% of males survived 3 weeks at least, although in the latter case more males died within 10 days after emergence (Figure A1). In the NyIM/HF group, at 60 Gy two peaks appear, the higher at about 30 days and the lower at about 1 week. The two peaks remain visible at higher doses, although with a slight tendency to life span reduction.

#### 3.3.2. Irradiated Males at Nymphal Stage or Late Adult Stage

Irradiation negatively affected survival (Table A4; deviance of the overall effects: *χ*^2^ = 1184.10, *p*-value ≤ 2.2 × 10^−16^ ***). The males collected at about 2 weeks after adult emergence showed a shorter life span in comparison to the males irradiated at the nymphal stage, as the experiment in the latter case was set up immediately after adult emergence (*t*-value = −2.976 **). No significant interaction effects were found, although the positive coefficients of the interaction terms suggested a lesser effect of the irradiation on the OIM/HF group than on the NyIM/HF group (Table A4).

No significant differences among doses were found in the OIM/HF adults (Table 5), even when compared with the untreated, indicating that increasing doses of gamma rays had a mild effect on their life span. The shorter life span of the untreated males of the OIM/HF group was mainly due to the late start of the experiment compared to the NyIM/HF group (for the untreated males: NyIM/HF: 51.04 ± 3.27 days; OIM/HF: 31.50 ± 6.93 days). However, males of the NyIM/HF group significantly reduced their life span as the dose increased (Table 5), approaching values of the OIM/HF males at 80 Gy (22.13 ± 3.35 days for NyIM/HF and 22.81 ± 2.61 days for OIM/HF) and at 100 Gy (18.71 ± 2.85 days for NyIM/HF and 17.79 ± 1.61 days for OIM/HF). Thus, the irradiation treatment had a significant impact on the NyIM/HF group (score (logrank) test = 53.92, 1 df, *p*-value = 3 × 10^−11^) but was weaker in the group of the “old” males (score (logrank) test = 4.16 on 1 df, *p*-value = 0.04).

**Figure A2:**
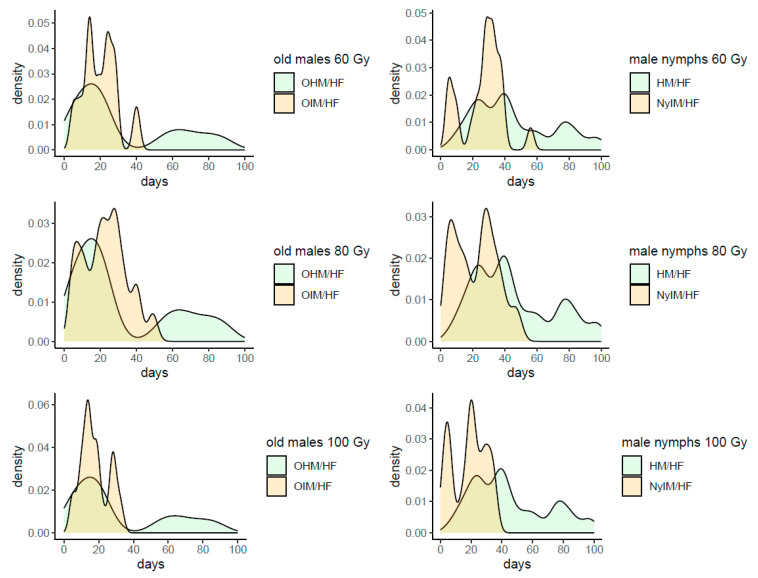
Kernel density plots of frequency. Comparison between the life span of the irradiated male nymphs (NyIM/HF) and the life span of the males irradiated two weeks after adult emergence (OIM/HF). The green curves represent the untreated control and are compared with the orange curves of the irradiated individuals at the doses of 60, 80 and 100 Gy.

As the Kernel density plots of frequency show (Figure A2), two groups are visible in the untreated control of the OIM/HF males with a different life span (a frequency peak at about 15 days and another smaller peak at about 60 days). A portion of about 30% of long-lived males with a life span of about 40 days was still present at 60 Gy. At 80 dose Gy, the highest peak of frequency was at about 25 days while at 100 Gy, the highest peak was at 15 days. In the NyIM/HF group, the highest peak of frequency was at about 30 days of life span, at 60 Gy, 25 days at 80 Gy and at about 20 days at 100 Gy, thus very close to the main peak of the OIM/HF cross. The difference between the two crosses can be found in the secondary peak of males with a very short life span (1 week or less) well visible in the NyIM/HF cross and almost absent in the OIM/HF cross (Figure A2).

## 4. Discussion

Based on the experience with another pentatomid, *H. halys*, critical problem facing SIT implementation on stink bugs is the impracticability of sustainable mass rearing. It has been proposed that this challenge could be mitigated by catching and rearing mature males of previously wild-harvested *B. hilaris* when they aggregate before the winter, with the idea of sterilizing and releasing them at the end of the overwintering diapause during the spring [24,28].The success of an area-wide pest management program that has the SIT as its strategic core relies on setting up a system to release sterile males able to compete with wild males for mating. Therefore, a recent study was focused on the selection of the optimal irradiation dose, able to induce sperm sterility in irradiated males while at the same time maintaining the mating competitiveness of the sterile bagrada bug males with respect to the fertile insects [42]. Given the difficulty in mass rearing of this species of stink bugs, the possibility of collecting large numbers of aggregating pre-diapause adults in autumn by extensive mass trapping and use of the sterile irradiated males for SIT small-scale approaches has been considered [24].

The main objective of the behavioral bioassays carried out here was to demonstrate that gamma irradiation is effective on some physiological parameters of fifth nymphal instars and mature adults of *B. hilaris*. Similar work was carried out a few years ago [26], showing that gamma rays were able to induce sterility in the sperm of newly emerged males, a first important result supporting application of the SIT to control this important alien pest.

In contrast to the results of the previous work [26], gamma irradiation (from 60 to 120 Gy for the adults and 32 to 100 Gy for fifth-instar nymphs) does not interfere with the longevity of adult females of *B. hilaris*, while having a moderate negative impact on the survival of the males (see Table 5). In any case, the longevity of irradiated mature males is still long enough to allow their release in the field for a SIT program: in fact, 50% of males survived at least 3 weeks or more when irradiated from 60 Gy upward. Particularly, a peak of frequency in life span of about 30 days was observed at 60 Gy, while at 80 and 100 Gy a portion higher than 50% of males survived 3 weeks at least, although in the latter case more males died within 10 days after emergence (Figure A2). Even if the test of longevity started 2 weeks after adult emergence, the remaining life span of the males irradiated at mature age was comparable with the longevity of the males irradiated at the nymphal stage, confirming that adult stage has a better capability to contrast the negative effects of gamma irradiation on this physiological aspect (Table 5, Figure A2).

There was a strong negative impact of irradiation dose on hatching rate (number of eggs that hatched), regardless of the physiological stage when bagrada bug males were irradiated (Table 2, Figure 4). When irradiation was applied on fifth-instar nymphs, the emerged females are showing an important decrease in fecundity (number of eggs laid; Figure 1). This result is similar to what was found previously when irradiation was applied to newly emerged adults [26]. Combining the results of the effects of irradiation on the longevity and on the fertility (hatching rate of the eggs of fertile females mated with irradiated males), the most suitable dose is confirmed to be 80 Gy, for both bagrada bugs irradiated as old adults or at the fifth nymphal instars. In fact, even if differences were recorded in the amount of irradiation to achieve full sterilization (respectively, 75 Gy for fifth-instar nymphs and 106 Gy for old adults, see Table 4), the effects of the irradiation on the longevity and the performance are supportive to use the irradiation dose at 80 Gy to reach the most correct trade-off to between sterility, longevity and mating performance in irradiated males [42].

The use of wild-type adults in biological control in general and SIT programs includes advantages and disadvantages compared to traditional mass rearing. Among the positive aspects, we can consider the following:

- Economic. Collecting insects of target species in the field instead of rearing them in huge bio-factory facilities should be less expensive, agronomically supportive as it reduces the pressure of the pest locally, environmentally friendly and, in some cases (i.e., gregarious insects), very easy and fast. At the end of autumn and at the beginning of winter (generally very mild in Pantelleria), mature bagrada nymphs and adults aggregate in huge clusters (from several hundreds to few thousands individuals each): the gregarious behavior in this period is more to create suitable physiological diapause conditions than to maximize the feeding (all the caper plants are without leaves). This would be the perfect period to perform the mass trapping. The mutual effect of removing large numbers of females from the environment and re-introduce sterile (irradiated at 80 Gy) wild-type males for small-scale SIT geo-localized applications, should play an important role in the management of this species, when infestations are very restricted like in the case of Pantelleria Island.
- Competitive. Wild-type collected insects should perform better than reared ones. In the case of mass rearing, one of the challenging requirement to successfully apply the SIT, is producing huge numbers of insects maintaining an adequate quality for the subsequent release in the field [43]. In the past, poor performance of sterile males in terms of mating competitiveness has been always attributed to side effects of irradiation [44,45] and was addressed by “overflooding” ratios; on the contrary, it has been demonstrated that the mass-rearing process can promote genetic drifts, selecting genotypic characteristics in laboratory populations more suitable for captivity [26].
- Conservative. The suggested approach (catch–irradiate–release) will not introduce new organisms in the area where it is applied, rather it will utilize wild males already in the field. This type of small-scale SIT could therefore be applied in areas like national parks, where the voluntary release of non-indigenous organisms (even if beneficial) is not allowed.
- Impactful. Field traps trigged with the aggregation pheromone or just massive collections during the autumn in the areas where *B. hilaris* is aggregating can provide large numbers of alive adults of both sexes: keeping (or eliminating) caught females and releasing wild males after irradiation in the environment can give more chances to the sterile males to find fertile females with whom to mate, removing at the same time the additional feeding impact induced by the massive release (according to the suitable overflooding ratio) of new adults reared in bio-factory facilities.

On the other hand, there are also some negative aspects around the use of wild-collected insects for the SIT:

- Pathogenic. Arthropods collected in nature could be affected by some pathogenic diseases, such as bacteria, fungi and protozoa. Even in cases where the infection rate at the beginning is very low, performing mass trapping and consequently confining large numbers of the collected insects in laboratory cages can cause the spreading of the infection to the full colony. An important mitigation would be to periodically preserve in ETOH 95% small samples of individuals from each site to detect the eventual presence of pathogens, keeping them separated in small cages to limit and manage the contamination effect.
- Marking. Unfortunately, *B. hilaris* is very sensitive to marking methods tested so far. For this reason, it is still not possible to mark the irradiated individuals to release in the open field, making it difficult to conduct early monitoring detection of the validity of the SIT program. Among the possible solutions to solve this issue, in addition to finding a suitable (easy and fast) marking system, is to verify the suitability of the system in semi-field conditions, starting from a known number of fertile individuals, detecting the eventual decrease in the population in the presence of sterile irradiated males.
- Logistically. The idea to build a structure similar to the Calliope Irradiation Unit at the ENEA Casaccia is unrealistic and not sustainable. SIT applications on a small scale need the use of irradiation sources alternative to the ^60^Co. One possible alternative is given by linear accelerators, producing high-energy photons and used in hospitals for medical purposes: they have been successfully tested with another pentatomid pest species, achieving a sterility level of 95% exposing *H. halys* males at a 32 Gy X-ray irradiation dose [28].

Considering the differences in terms of fertility and fecundity, we can hypothesize that the “holistic” irradiation of wild-type *B. hilaris*, followed by the release of sterile bagrada bugs at different physiological stages and of both sexes, has a good chance to be successful as an SIT program suppressing the target populations of bagrada bugs. This is before considering the additive effect with biological control by the use of egg parasitoids, which should increase the effectiveness of control. An integrated approach, combining the SIT and biological control, could be particularly helpful because fertile females mating with irradiated males will lay the same number of eggs (no interference with the fecundity, see Table A2) but the eggs will be sterile. Similarly to what has been proved on the brown marmorated stink bug (*H. halys*) [27], those “sterile” sentinel eggs could be a suitable substrate for the oviposition and full larval development of the egg parasitoid *G. aetherium* [46].

## 5. Conclusions

In conclusion, the results obtained in this study therefore provide a valuable starting point for implementing a new approach to use the aggregation behavior of *B. hilaris* during the autumn as a suitable field-harvest mass trapping tool to obtain huge amounts of adults to use for localized small-scale SIT applications alone and/or in combination with classic biological using egg parasitoids. Further field and semi-field tests are still needed, though, to assess the real feasibility of this potential IPM strategy.

## Figures and Tables

**Table 1:** Experiment on male and female adults emerged from late-stage irradiated nymphs. Effects on adult reproduction. Means and standard errors of the reproductive parameters are reported for each tested dose. HM/NyIF: irradiated females x healthy males; NyIM/HF: irradiated males × healthy females. Values followed from different letters are significantly different at *p* = 0.05 (Dunn test); letters refer to the comparisons performed within each group. Asterisks indicate a significant difference between the two treatments at *p* = 0.05 or at *p* = 0.001 (Mann–Whitney test).

	Dose (Gy)	HM/NyIF			NyIM/HF	
Eggs oviposited	0	150.72 ± 13.65	a		154.54 ± 12.45	a
	32	10.50 ± 3.07	b	***	150.50 ± 20.12	a
	40	9.90 ± 1.83	b	***	149.62 ± 16.99	a
	60	2.15 ± 0.82	c	***	174.96 ± 15.12	a
	80	1.50 ± 0.86	c	***	119.67 ± 20.34	ab
	100	-			75.79 ± 13.24	b
Eggs eclosed	0	97.14 ± 11.77	a		98.91 ± 11.57	a
	32	1.10 ± 0.90	b	***	29.88 ± 7.08	b
	40	0.14 ± 0.10	b	***	14.90 ± 3.07	b
	60	0.04 ± 0.04	b	*	2.35 ± 1.18	c
	80	0.14 ± 0.14	b		0.47 ± 0.35	c
	100	-			0.00 ± 0.00	c
% Eclosed	0	58.21 ± 4.05	a		59.31 ± 4.46	a
	32	4.82 ± 3.33	b		16.88 ± 2.66	b
	40	1.25 ± 0.95	b	*	8.99 ± 1.49	b
	60	0.71 ± 0.71	b		1.45 ± 0.59	c
	80	9.52 ± 9.52	b		0.62 ± 0.45	c
	100	-			0.00 ± 0.00	c

**Table 2:** Comparison among males irradiated at 3 different phases of development: irradiation dose applied to late-stage male nymphs, newly emerged males and old adult males. Effects on adult reproduction. Means and standard errors of the reproductive parameters are reported for each tested dose. NyIM/HF: males irradiated at the nymphal stage; NEIM/HF: newly emerged irradiated males; OIM/HF: males irradiated after about two weeks from adult emergence. Values followed from different letters are significantly different at *p* = 0.05 (Dunn test); small letters refer to the comparisons among doses performed within each treatment. Capital letters refer to the comparisons among treatments performed within each dose.

	Dose (Gy)	NyIM/HF		NEIM/HF		OIM/HF	
Eggs oviposited	0	154.5 ± 12.45	a A	129.9 ± 21.95	a A	141.9 ± 18.21	a A
	16	-		129.4 ± 21.44	a	-	
	24	-		117.0 ± 17.78	a	-	
	32	150.5 ± 20.12	a	111.8 ± 20.16	a	-	
	40	149.6 ± 16.99	a	-		-	
	50	-		142.6 ± 24.52	a	-	
	60	172.1 ± 15.56	a A	166.7 ± 42.83	a A	134.0 ± 9.93	ab A
	80	119.7 ± 20.34	ab AB	206.2 ± 37.82	a A	86.52 ± 11.02	b B
	100	75.79 ± 13.24	b A	105.2 ± 22.61	a A	96.75 ± 10.52	ab A
	120	-		171.6 ± 48.02	a	154.8 ± 19.93	a
	140	-		150.6 ± 32.09	a	-	
Eggs eclosed	0	98.91 ± 11.57	a A	82.08 ± 15.66	a A	88.17 ± 15.15	a A
	16	-		49.00 ± 11.12	ab	-	
	24	-		33.67 ± 7.53	ac	-	
	32	29.88 ± 7.08	b	29.40 ± 8.94	ac	-	
	40	14.90 ± 3.07	b	-		-	
	50	-		15.20 ± 3.41	ad	-	
	60	2.45 ± 1.22	c B	9.75 ± 4.82	bd AB	12.42 ± 2.53	b A
	80	0.47 ± 0.35	c B	15.25 ± 7.16	ad A	1.10 ± 0.371	c B
	100	0.00 ± 0.00	c A	0.67 ± 0.29	d A	1.75 ± 0.575	c A
	120	-		0.60 ± 0.60	cd	2.45 ± 0.717	c
	140	-		2.40 ± 1.75	cd	-	
% Eclosed	0	59.31 ± 4.46	a A	60.35 ± 6.02	a A	60.56 ± 6.28	a A
	16	-		37.35 ± 5.11	ab	-	
	24	-		31.59 ± 11.2	ab	-	
	32	16.88 ± 2.66	b	24.34 ± 4.30	ac	-	
	40	8.994 ± 1.49	b	-		-	
	50	-		13.52 ± 4.27	ad	-	
	60	1.52 ± 0.61	c B	6.242 ± 3.45	bd AB	8.69 ± 1.58	b A
	80	0.62 ± 0.45	c B	7.451 ± 3.95	bd A	1.26 ± 0.39	c B
	100	0.00 ± 0.00	c B	0.741 ± 0.34	d AB	2.30 ± 0.93	c A
	120	-		0.759 ± 0.76	cd	1.13 ± 0.28	c
	140	-		1.50 ± 0.93	cd	-	

**Table 3:** Comparison among males irradiated at 3 different phases of development: irradiation dose applied to late-stage male nymphs, newly emerged males and old adult males. Model fitted for the % of response in eggs hatching with a Weibull model. Upper limits *d* and lower limits *c* of response are shared among crosses (crossings of *Bagrada hilaris*: NyIM/HF: males irradiated at the stage of nymph; NEIM/HF: newly emerged males; OIM/HF: males irradiated after about two weeks from adult emergence. The parameter *b* describes the slope and the parameter *e* is the inflection point of the curve. Values followed from different letters are significantly different at *p* ≤ 0.05 according to the *t* test.

	Estimate		±S.E.	*t*-Value	*p*-Value
*b*:(Intercept)	1.3772		0.204	6.756	8.24 × 10^−11^ ***
*d*:(Intercept)	95.868		3.598	26.64	<2.2 × 10^−16^ ***
*e*: NyIM/HF	24.767	a	2.420	10.23	<2.2 × 10^−16^ ***
*e*: NEIM/HF	35.577	b	3.805	9.350	<2.2 × 10^−16^ ***
*e*: OIM/HF	35.074	b	4.231	8.289	4.87 × 10^−15^ ***

Levels of significance are reported in the table according to the conventional notation by asterisks: no symbols, *p* > 0.05; *p* ≤ 0.05 *; *p* ≤ 0.01 **; *p* ≤ 0.001 ***.

**Table 4:** Comparison among males irradiated at three different phases of development. Estimated effective dose (ED) calculated for the percentage of response in eggs hatching on three groups of crosses: NyIM/HF: males irradiated at the nymphal stage; NEIM/HF: newly emerged irradiated males; OIM/HF: males irradiated after about two weeks from adult emergence. Standard error and error limits at the 95 percentile are reported.

	ED (Gy)	Estimate	±S.E.	Lower	Upper
NyIM/HF:	50	18.980	2.4881	14.082	23.878
NyIM/HF:	90	45.382	2.7744	39.921	50.843
NyIM/HF:	95	54.938	3.9763	47.110	62.765
NyIM/HF:	99	75.070	7.9213	59.477	90.663
NEIM/HF:	50	27.260	3.5031	20.369	34.160
NEIM/HF:	90	65.190	6.9817	51.447	78.934
NEIM/HF:	95	78.917	9.6053	60.009	97.825
NEIM/HF:	99	107.84	16.609	75.142	140.53
OIM/HF:	50	26.879	4.1568	18.696	35.061
OIM/HF:	90	64.268	4.4577	55.493	73.043
OIM/HF:	95	77.800	5.5408	66.893	88.707
OIM/HF:	99	106.31	10.053	86.522	126.10

**Table 5:** Days of survival generated from Kaplan–Meyer curves recorded on the 3 tested groups of individuals: irradiated at late nymphal stage (male: NyIM/HF; females: HM/NyIF) and at two weeks after adult emergence (males: OIM/HF): number of records, means, standard errors of means, medians and lower and upper confidence limits at 95% are reported. Values followed from different letters are significantly different at *p* = 0.05 according to the Dunn test; letters refer to the comparisons performed within each group.

	n	Mean	±S.E.	Median	Lower	Upper	
females 0 Gy HM/HF	84	38.50	1.88	33.0	33	41	a
females 32 Gy HM/NyIF	10	25.30	3.19	23.0	18	32	b
females 40 Gy HM/NyIF	21	35.38	2.92	33.0	28	47	ab
females 60 Gy HM/NyIF	23	30.13	2.39	28.0	23	33	ab
females 80 Gy HM/NyIF	14	36.79	3.84	36.5	24	56	ab
females 100 Gy HM/NyIF	9	35.44	2.57	34.0	28	41	ab
males 0 Gy HM/HF	84	51.04	3.27	42.0	38	56	a
males 32 Gy NyIM/HF	16	34.50	5.29	28.0	14	39	ab
males 40 Gy NyIM/HF	21	29.14	3.61	28.0	18	47	b
males 60 Gy NyIM/HF	23	26.39	2.60	28.0	23	33	b
males 80 Gy NyIM/HF	15	22.13	3.35	25.0	12	35	b
males 100 Gy NyIM/HF	14	18.71	2.85	20.0	14	25	b
males 0 OHM/OHF	16	31.50	6.93	18.5	14	69	a
males 60 OIM/HF	24	20.00	1.88	19.0	14	24	a
males 80 OIM/HF	21	22.81	2.61	23.0	17	33	a
males 100 OIM/HF	24	17.79	1.61	15.5	14	23	a

## Data Availability

The data presented in this study are available on request from the corresponding author.

## Appendix A

**Table A1:** Experiment on male and female adults emerged from late-stage irradiated nymphs: coefficient estimates of the GLiM models for the effects of irradiation dose on the number of eclosed eggs, the number of oviposited eggs and the proportion of eggs eclosed per female. The HM/NyIF cross does not appear in the table since this parameter is set to zero and matters as a reference level; thus, the estimates are compared with this group.

Response Variable	Levels	Estimate	±S.E.	*t*-Value	*p*-Value
	intercept	4.932	0.1153	42.77	<2 × 10^−16^ ***
Eggs oviposited	dose	−0.065	0.0030	−21.56	<2 × 10^−16^ ***
	NyIM/HF	0.210	0.1567	1.342	0.18
	dose: NyIM/HF	0.061	0.0036	16.97	<2 × 10^−16^ ***
	intercept	4.599	0.2628	17.50	<2 × 10^−16^ ***
Eggs eclosed	Dose	−1.582	0.1081	−14.64	<2 × 10^−16^ ***
	NYIM/HF	0.281	0.3725	0.755	0.45
	dose: NyIM/HF	0.838	0.1331	6.299	3 × 10^−16^ ***
	intercept	3.4829	0.1747	19.94	<2 × 10^−16^ ***
Proportion of eclosed eggs	dose	−0.061	0.0050	−12.12	<2 × 10^−16^ ***
	NyIM/HF	0.236	0.2371	0.995	0.32088
	dose: NyIM/HF	0.017	0.0058	2.982	0.00323 **

Levels of significance are reported in the table according to the conventional notation by asterisks: no symbols, *p* > 0.05; *p* ≤ 0.05 *; *p* ≤ 0.01 **; *p* ≤ 0.001 ***.

**Table A2:** Comparison among males irradiated at 3 different phases of development: effects of irradiation dose applied to late-stage male nymphs, newly emerged males and old adult males on adult reproduction. Coefficient estimates of the GLiM models for the number of eclosed eggs, the number of oviposited eggs and the proportion of eggs eclosed per female. Types of crosses: NEIM/HF: newly emerged irradiated males; OIM/HF: males irradiated after about two weeks from adult emergence. The NyIM/HF cross (adults males from irradiated nymphs) does not appear in the table since this parameter is set to zero and matters as a reference level; thus, the estimates are compared with this group.

Response Variable	Levels	Estimate	±S.E.	*t*-Value	*p*-Value
	intercept	5.1107	0.0778	65.70	<2 × 10^−16^ ***
Eggs oviposited	dose	−0.0038	0.0015	−2.591	0.0101 *
	NEIM/HF	−0.2458	0.1600	−1.537	0.1257
	OIM/HF	−0.2413	0.1411	−1.710	0.0885
	dose: NEIM/HF	0.0050	0.0024	2.086	0.0380 *
	dose: OIM/HF	0.0030	0.0020	1.452	0.1478
	intercept	4.6193	0.0736	62.77	<2 × 10^−16^ ***
Eggs eclosed	dose	−0.0513	0.0048	−10.67	<2 × 10^−16^ ***
	NEIM/HF	−0.2041	0.1509	−1.352	0.1775
	OIM/HF	−0.1378	0.1324	−1.041	0.2991
	dose: NEIM/HF	0.0182	0.0071	2.573	0.0107 *
	dose: OIM/HF	0.0147	0.0058	2.538	0.0118 *
	intercept	3.7390	0.1385	27.00	<2 × 10^−16^ ***
Proportion of eclosed eggs	dose	−0.0432	0.0024	−17.70	<2 × 10^−16^ ***
	NEIM/HF	0.2864	0.2642	1.084	0.2794
	OIM/HF	−0.1885	0.2330	−0.809	0.4191
	dose: NEIM/HF	0.0097	0.0040	2.443	0.0153 *
	dose: OIM/HF	0.0149	0.0032	4.532	9.17 × 10^−16^ ***

Levels of significance are reported in the table according to the conventional notation by asterisks: no symbols, *p* > 0.05; *p* ≤ 0.05 *; *p* ≤ 0.01 **; *p* ≤ 0.001 ***.

**Table A3:** Experiment on males and female adults emerged from late-stage irradiated nymphs: coefficient estimates of the GLiM models for the effects of irradiation dose on the life span of males and females. Dose zero and females do not appear in the table since these parameters are set to zero and matter as reference levels.

	Estimate	±S.E.	*t*-Value	*p*-Value
intercept	3.6507	0.0555	65.76	<2 × 10^−16^ ***
32 Gy	−0.4199	0.2061	−2.037	0.042450 *
40 Gy	−0.0845	0.1284	−0.658	0.511141
60 Gy	−0.2451	0.1322	−1.855	0.064528
80 Gy	−0.0456	0.1498	−0.304	0.761248
100 Gy	−0.0827	0.1853	−0.446	0.655660
males	0.2819	0.0735	3.833	0.000152 ***
32 Gy: males	0.0283	0.2507	0.113	0.910236
40 Gy: males	−0.4758	0.1874	−2.540	0.011569 *
60 Gy: males	−0.4144	0.1903	−2.178	0.030159 *
80 Gy: males	−0.7899	0.2341	−3.375	0.000829 ***
100 Gy: males	−0.9206	0.2733	−3.368	0.000848 ***

Levels of significance are reported in the table according to the conventional notation by asterisks: no symbols, *p* > 0.05; *p* ≤ 0.05 *; *p* ≤ 0.01 **; *p* ≤ 0.001 ***.

**Table A4:** Experiment on males irradiated at 2 different phases of development: coefficient estimates of the GLiM model for the effects of irradiation dose on the life span of males irradiated as nymphs (NyIM/HF) or as two-week-aged adults (OIM/HF). The dose zero and the NyIM/HF cross do not appear in the table since these parameters are set to zero and matter as reference levels.

	Estimate	±S.E.	*t*-Value	*p*-Value
intercept	3.9325	0.0526	74.777	<2 × 10^−16^ ***
60 Gy	−0.6595	0.1493	−4.416	1.60 × 10^−5^ ***
80 Gy	−0.8354	0.1962	−4.259	3.08 × 10^−5^ ***
100 Gy	−1.0032	0.2191	−4.578	7.97 × 10^−6^ ***
OIM/HF	−0.4825	0.1622	−2.976	0.00326 **
60 Gy: OIM/HF	0.2052	0.2656	0.773	0.44049
80 Gy: OIM/HF	0.5126	0.2946	1.740	0.08323
100 Gy: OIM/HF	0.4320	0.3151	1.371	0.17189

Levels of significance are reported in the table according to the conventional notation by asterisks: no symbols, *p* > 0.05; *p* ≤ 0.05 *; *p* ≤ 0.01 **; *p* ≤ 0.001 ***.
